# Optimization of microwave-assisted extraction for quercetin (prebiotic) and the effect of its symbiotic combination with *Lactobacillus acidophilus* (probiotic) in NAFLD induced rat model

**DOI:** 10.3389/fnut.2025.1596758

**Published:** 2025-06-20

**Authors:** Mehwish Majeed, Waqas Ahmed, Sumera Javad, Iahtisham-Ul-Haq, Summer Rashid, Rashida Perveen, Umar Farooq, Juweria Abid, Abdul Momin Rizwan Ahmad

**Affiliations:** ^1^Department of Food Science and Human Nutrition, University of Veterinary and Animal Sciences, Lahore, Pakistan; ^2^Department of Botany, Lahore College for Women University, Lahore, Pakistan; ^3^Kauser Abdulla Malik School of Life Sciences, Forman Christian College (A Chartered University), Lahore, Pakistan; ^4^School of Food Science and Technology, Minhaj University Lahore, Lahore, Pakistan; ^5^Department of Human Nutrition and Food Technology, Faculty of Allied Health Sciences, Superior University Lahore, Lahore, Pakistan; ^6^Department of Nutrition and Dietetics, National University of Medical Sciences (NUMS), Rawalpindi, Pakistan; ^7^Department of Health Sciences, University of York, York, United Kingdom; ^8^Department of Human Nutrition and Dietetics, NUST School of Health Sciences, National University of Sciences and Technology (NUST), Islamabad, Pakistan

**Keywords:** microwave-assisted extraction, response surface methodology, quercetin, *Lactobacillus acidophilus*, non-alcoholic fatty liver disease

## Abstract

**Introduction:**

Changing dietary patterns, lifestyle related disorders and associated metabolic syndromes have increased the prevalence of NAFLD over the last few years. It has been observed that there is a direct association between intestinal dysbiosis and NAFLD truly depicted by interconnected complex mechanisms. Besides its antioxidant activity, quercetin serves prebiotic functions as well.

**Objective:**

The objective of the current research was to determine the synbiotic effect of quercetin and *Lactobacillus acidophilus* on non-alcoholic fatty liver disease (NAFLD) induced rat models.

**Methods:**

Quercetin was extracted from red onions via microwave-assisted extraction technique (MAE). Response Surface Methodology (RSM) was employed to optimize MAE parameters. 25 female albino rats were divided into 5 groups of 5 rats each; 2 control (untreated and negative control) and 3 treatment groups (G1, G2, G3). High fat diet (HFD) (40% fat) in combination with 15% sucrose water and 440 mg cholesterol/100 g feed was given to rats over a period of 6 weeks to induce NAFLD. For the efficacy trial, treatment groups received different doses of quercetin; 50 mg, 80 mg and 100 mg in G1, G2 and G3, respectively, with a dose of 10^2^ CFU of *Lactobacillus acidophilus*/200 μL of PBS in all three groups.

**Results:**

The results revealed optimal MAE conditions for maximum amount of quercetin as 600 W microwave power, 3 min irradiation time and distilled water as a solvent. Resultantly, 86.10 mg quercetin/gram of red onion extract (32.7mgQ/g onion powder) was obtained. There was no significant difference in HDL, VLDL, triglycerides, serum AST and serum ALP levels (*p*-value > 0.05) between all groups. However, total cholesterol, LDL cholesterol and serum ALT significantly improved in G3 (*p*-value < 0.05).

**Conclusion:**

The synbiotic combination is effective at lowering total cholesterol, LDL cholesterol as well as serum ALT levels at a dose of 100 mg of quercetin/kg body weight for rats.

## Graphical abstract

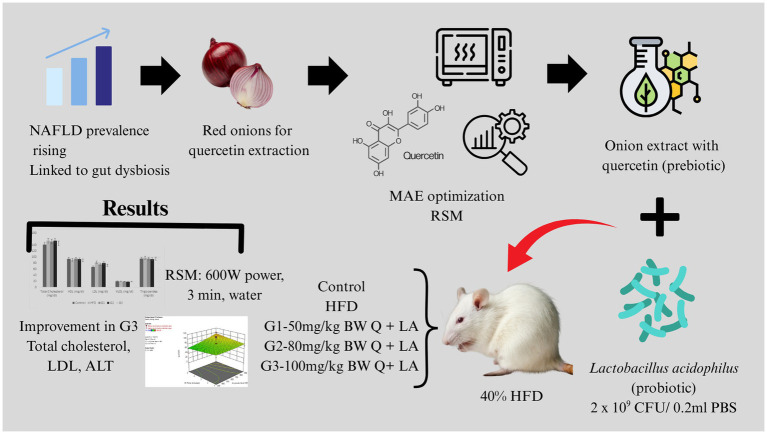

## 1 Introduction

Non-alcoholic fatty liver disease (NAFLD) has become one of the most common hepatic diseases worldwide with a global prevalence of 32.4% (1). According to one study in United States, the rise in NAFLD prevalence is linked to an increase in liver mortality (2). Apart from the western world, NAFLD is on rise in Asian countries as well. Prevalence has increased dramatically since 1999 with overall mortality rate of 5.3 deaths/1,000 person-years (3). Due to the rise in obesity and unhealthy lifestyle, Pakistan is also at stake of increased hepatic mortality related to NAFLD. Among the general population of Pakistan, 15% people are at risk to have fatty liver (4).

NAFLD begins with accumulation of fat in liver cells (steatosis) leading to lipotoxicity which may progress toward steatohepatitis (NASH), liver fibrosis and ultimately hepatic carcinoma (5). It is suggested that a combination of variables, including dietary factors, insulin resistance, obesity, genetic and epigenetic factors and gut microbiota leads to the progression of the disease (6). Over the last decade, there have been numerous research instances on role of gut microbiota and their associated claims in maintaining overall physiology. Additionally, role of microbiota has been implicated in their roles against progression in type II diabetes, obesity and its resultant effects leading to onset of NAFLD (7). Mechanistically, quercetin and similar polyphenols can serve as substrates for microbial metabolism, leading to the production of short-chain fatty acids (SCFAs), especially butyrate, which supports colonocyte health and modulates immune responses (8). Additionally, polyphenol-induced shifts in microbial composition can influence host metabolic pathways, contributing to anti-obesity and anti-diabetic effects.

Gut microbiota functions as a separate metabolic organ in the body. Its major role is the fermentation of carbohydrates and the production of short chain fatty acids (SCFAs). Recently it has been found that imbalance in the gut microbiota enhances intestinal permeability to gut microbes as well as the exposure of liver to toxic substances promoting lipogenesis and fibrosis (9).

Given the lack of an efficient therapy, the main strategy for the management of NAFLD is to shift sedentary lifestyle to an active, healthy one; with nutritional intervention being one of the primary approaches (10). However, it has been observed that adherence to such type of interventions is difficult for patients. So, there is a need to search for new effective treatment strategies for the disease. As a result, administration of probiotics or prebiotics can be suggested as a viable treatment option for the management of NAFLD since alterations in the gut microbiota plays a significant role in the development of NAFLD (9). Furthermore, the synbiotic combination, i.e., the combination of both prebiotics and probiotics may pose a synergistic effect and increase the effectiveness of the intervention (11). In this lieu, several probiotics spp. like *Lactobacillus rhamnosus* and *Bifidobacterium longum* have been shown to impart reduction in liver fat accumulation and serum ALT levels in both animal models and clinical studies (12). Besides, there are certain other instances in which sufficient data report associated health promoting potential of probiotics like *Lactobacillus plantarum* and *Lactobacillus casei.*

Quercetin, a flavonoid, is found in a variety of plants. It is produced by more than 20 plant species, most abundantly found in apples, onions and wines, with some quantity in raddish, coriander and dill (13). However, onions have been shown to possess the highest quantity of quercetin with an approximate amount of 300 mg/kg (14).

In the recent years, novel extraction techniques have been extensively studied in an effort to extract valuable natural compounds from plants. Microwave-assisted extraction (MAE) is a green technology and an effective method for employing shorter extraction time, less solvent consumption and securing thermolabile components from degradation (15).

In Mae, the sample is directly heated using microwaves which result in efficient extraction of target compound. This increases the solubility and diffusion of that compound in the solvent. In this way, less solvent is required for extraction making this technique environment friendly and cost effective (16). Studies comparing traditional Soxhalet extraction to MAE revealed MAE used 79 times less energy as Soxhalet, requires less solvent and extraction time and even outperforms other extraction techniques (supercritical fluid extraction, Ultrasound extraction technique, pressurized liquid extraction) in terms of total phenolic content (17–19). Therefore, MAE can be suitable choice for quercetin extraction for further study.

Quercetin has long been studied for its anti-inflammatory and antioxidant properties. It has been shown to ameliorate symptoms of metabolic syndrome and NAFLD by improving insulin sensitivity, reducing buildup of lipids in liver and by modulating certain lipogenic genes in *in vivo* models. However, recent studies reveal that quercetin possesses a potential prebiotic effect in addition to its antioxidant and anti-inflammatory properties (20).

Moreover, the probiotic *Lactobacillus acidophilus* has also been identified to affect the development of NAFLD and is proposed as a useful treatment strategy. It has been shown to reduce body weight, liver steatosis and also improves serum cholesterol levels (21).

It has been observed that contemporary research on synbiotic combinations as an adjunct therapy against NAFLD is scarce and is in the emerging phase. Keeping in view the abovementioned claims, the present study is designed to validate the combined effect of nutritional interventions along with synbiotic combination of quercetin and *Lactobacillus acidophilus*. Resultantly, impact of cumulative administration of quercetin and *Lactobacillus acidophilus* against NAFLD progression and its associated symptoms is limelight of the study. Additionally, the present study largely focuses on optimization of MAE parameters for effective quercetin extraction from red onions and its resultant impact against NAFLD and allied biomarkers.

## 2 Materials and methods

### 2.1 Raw materials

The study was performed at the Department of Botany, Lahore College for Women University, Lahore and the Department of Food Science and Human Nutrition, University of Veterinary and Animal Sciences, Lahore. Red onions (for the extraction of Quercetin) were purchased from local market. Single strain culture of *Lactobacillus acidophilus* was procured from SACCO® industries sold under the market name Lyofast LA3. Animal fat, used to prepare high fat diet, was also purchased from the local market. All other chemicals used in the trial were obtained from Sigma-Aldrich. Likewise, HPLC grade chemicals were purchased for subsequent HPLC assay.

### 2.2 Quercetin extraction

#### 2.2.1 Onion powder

Red skinned onions (*Allium cepa L*.) purchased from the local vegetable market were processed to remove any dirt and debris. Afterwards, the bulbs were peeled to remove skin and apical stems. Onion flesh was then diced into thin slices and was left to air dry overnight. After that, the semi dried onion slices were placed in a drying oven at 60°C for 72 h. The final dried onions were then pulverized into a fine powder using an electric lab grinder. The obtained powder was stored in an air tight plastic bag until further use.

#### 2.2.2 Experimental design

The extraction was done through microwave-assisted extraction (MAE) technique. Response surface methodology (RSM) was used to optimize extraction parameters using a central composite design (CCD) as shown in Table 1.

**Table 1:** Level of parameters for experimental design in MAE.

Microwave parameter	Unit	Code	Levels	
			Low (−)	High (+)
Power	Watt	A	300	900
Time	Minutes	B	1	5
Solvent	-	C	-	-

CCD was applied because it allows us to investigate the effect of multiple variables on the extraction of focus bioactive components and also to find the optimal conditions for their maximum extraction. Using Design Expert Software version 13.0, three microwave parameters (independent variables); solvent (ethanol/water), time of irradiation (minutes) and microwave power (watt) were studied for two responses; amount of extract (grams) and amount of Quercetin (mg of Q/g of extract). Levels of parameters used were selected on the basis of literature review (Table 1).

#### 2.2.3 Extraction procedure

Extraction was performed in a domestic microwave oven using a fixed solute to solvent ratio of 1:10 (1 g of onion powder in 10 mL of solvent) for each extraction. In this fashion, 26 extractions were carried out with their respective solvent, irradiation time and microwave power as given in Table 2. After the extraction, the extracts were filtered using Whattman filter paper and transferred to pre-weighed vials. The extracts were then left to air dry at room temperature for 24 h. Once the extracts have dried completely, the vials were then capped and stored in refrigerator until further analysis.

**Table 2:** Central composite design (CCD) for MAE variables; Microwave Power (A), Irradiation time (B), Solvent (C).

Independent variables			
Run no.	Power (W) (A)	Time (min) (B)	Solvent (C)
1	600	3	Ethanol
2	1024.26	3	Ethanol
3	900	1	Water
4	600	0.17	Ethanol
5	600	5.83	Water
6	600	3	Water
7	900	5	Ethanol
8	600	3	Ethanol
9	300	1	Ethanol
10	600	3	Water
11	300	5	Ethanol
12	175.74	3	Ethanol
13	300	1	Water
14	600	3	Ethanol
15	1024.26	3	Water
16	600	3	Water
17	900	5	Water
18	300	5	Water
19	600	3	Ethanol
20	600	3	Water
21	175.74	3	Water
22	900	1	Ethanol
23	600	5.83	Ethanol
24	600	3	Ethanol
25	600	0.17	Water
26	600	3	Water

### 2.3 Characterization of extract

#### 2.3.1 Flavonoid assay

Total Flavonoid Content (TFC) of the onion extract was estimated by following the method devised by Csepregi et al. (22) with some changes. For the assay, onion extracts were prepared by dissolving 1 mg of extract in 1 mL distilled water to obtain a concentration of 1 mg/mL. To prepare the solution, 1.25 mL of distilled water was added in the extracts. Following this, 75 μL of 5% sodium nitrate (NaNO3) solution was added. After 5 min, 150 μL of 10% aluminum chloride (AlCl3) solution was added into the extract solutions. After another minute, 500 μL of 1 M sodium hydroxide (NaOH) solution was added. Finally, 275 μL of distilled water was added and the resulting solution was stirred thoroughly. Before spectrophotometry, reaction precipitates were filtered using syringe filters of 200 micrometer pore size. Absorbance of the clear solution was measured at 365 nm using a UV–Visible spectrophotometer.

#### 2.3.2 High performance liquid chromatography with ultraviolet

Based on the results of the experiment, selected extracts were analyzed for the amount of quercetin using high performance liquid chromatography. High performance liquid chromatography with ultraviolet (HPLC-UV) was performed at a commercial level at University Diagnostic Lab, Department of Microbiology, UVAS. A reversed phase C-18 column was used. Mobile phase consisted of acetonitrile, 0.3% trifluoroacetic acid and methanol in a ratio of 30:50:20, respectively. Detection was performed at a UV range of 254–368 nm.

### 2.4 Experimental animals and diet

25 females, albino rats, aged 3 weeks were bought and housed in an animal lab at the Institute of Biochemistry, University of Veterinary and Animal Sciences, Lahore. Rats were kept under standard conditions of 20–25°C temperature and 12 h’ light–dark cycle. Rats were treated according to the standard lab animal care and use guidelines. The study was approved by the Ethical Review Committee of the University of Veterinary and Animal Sciences, Lahore, Pakistan vide letter no. DR/249.

An acclimation period of 2 weeks was observed prior to the study. All rats were fed a standard diet and were randomly divided into 5 groups of 5 rats each. 2 control groups (Control, HFD) and 3 treatment groups (G1, G2, G3) were made on the basis of their diet. The control group received standard diet throughout the study whereas the control negative (HFD) and all other treatment groups (G1, G2, G3) were fed a combination diet for 6 weeks to induce NAFLD. High fat diet (HFD) in combination with sucrose water and high cholesterol was used to induce NAFLD. Rats were fed with a diet containing 40% animal fat, 15% sucrose water and cholesterol in a proportion of 440 mg/100 g of feed for 6 weeks. After 6 weeks, blood samples were taken to confirm the onset of disease. Raised levels of serum ALT and AST were considered as the markers of NAFLD.

After NAFLD has been successfully induced, all treatment groups were fed standard diet till the end of the trial.

### 2.5 Efficacy trial

An efficacy trial of 3 weeks was run to check the efficacy of the synbiotic combination of Quercetin (prebiotic) and *Lactobacillus acidophilus* (probiotic) against NAFLD. Doses of quercetin and *Lactobacillus acidophilus* are given in Table 1. Doses have been established on the basis of literature review. All treatment doses were administered using oral gavage technique.

### 2.6 Collection of samples

Blood samples were collected once after 6 weeks and then again at the end of the experimental study to check efficacy of treatment. Blood sampling was done under chloroform anesthesia via cardiac puncture technique. For biochemical analysis, blood was stored in clot activator vacutainers to separate serum. At the end of the experimental study, animals were euthanized via cervical dislocation under chloroform anesthesia and dissection was performed. For histopathological analysis, liver tissues were removed immediately and dipped in chilled normal saline. Afterwards, tissues were marked, labeled and stored in formalin solution until further analysis.

### 2.7 Biochemical analysis

Serum Lipid profile parameters *viz*; serum triglycerides, total cholesterol, LDL cholesterol, HDL cholesterol, and VLDL cholesterol, were determined using commercial kits bought from Sigma-Aldrich. Absorbance was measured at 507/650 nm using double beam spectrophotometer and the results were represented in mg/dl.

Similarly, liver function parameters; alanine transaminase (ALT), aspartate transaminase (AST), and alkaline phosphatase (ALP), were also assessed using the kit method (Sigma-Aldrich).

### 2.8 Liver histopathology

After animal dissection, liver tissue was fixed in neutral buffered formalin solution. For slide preparation, formaldehyde-fixed tissue was first hydrated, dehydrated, dipped in chloroform and xylene and then fixed in paraffin wax. A rotary microtome was used to cut 5 mm thickness sections of the tissue and left overnight at room temperature. The sections then underwent rehydration via a declining alcohol series (100, 90, 70, 50%) and washed with distilled water. The rinsed sections were then passed through an increasing series of alcohol after being stained with Hematoxylin and Eosin (H&E) dyes. Morphology of the tissue was examined using a microscope.

### 2.9 Statistical analysis

Data obtained in the 1st phase of study were analyzed using Design Expert® (DX) software version 13.0 whereas the Statistical Package for Social Sciences (SPSS) IBM, SPSS Inc., version 23.0 was used to analyze data from the 2nd phase of the study. One-way Analysis of Variance (ANOVA) was applied to compare means of all groups. *p* value less than 0.05 was considered significant. LSD *post hoc* test was used to perform intergroup comparisons.

## 3 Results

### 3.1 Quercetin extraction

#### 3.1.1 Onion powder

On an average, 1 kg of onions yielded almost 103.33 ± 8.819 g of dried onion powder. A total of 26 g powder was used for optimization procedure whereas 200 g onion powder was used for batch extraction of quercetin for animal dose preparation.

#### 3.1.2 Outcome of experimental design

In the present study, experimental design (as shown in Tables 1, 2) was employed to investigate the relationship between dependent and independent variables. A total of 26 experimental runs were performed according to the experimental design given in Tables 1, 2. Two responses, i.e., amount of extract and amount of quercetin were recorded for each run (Table 3). These responses were then analyzed using analysis of variance (ANOVA) for Quadratic model with Design Expert Software® (Table 4).

**Table 3:** CCD responses of MAE variables; amount of extract (g), amount of quercetin (μg of quercetin per mg of extract).

	Variables			Responses	
Run no.	Power (W) (A)	Time (min) (B)	Solvent (C)	Amount of extract (g)	Amount of quercetin (μg of Q/mg of extract)
**1**	**600**	**3**	**Water**	**0.19**	**109.38** ^**^
2	1024.26	3	Ethanol	0.03	90.04
3	900	1	Water	0.45	23.82
4	600	0.17	Ethanol	0.13	88.93
5	600	5.83	Water	0.27	32.27
**6**	**600**	**3**	**Ethanol**	**0.38**	**15.16** ^**^
7	900	5	Ethanol	0.27	99.82
8	600	3	Ethanol	0.12	58.27
9	300	1	Ethanol	0.14	67.16
10	600	3	Water	0.32	37.82
11	300	5	Ethanol	0.37	46.04
12	175.74	3	Ethanol	0.12	79.16
13	300	1	Water	0.16	26.27
14	600	3	Ethanol	0.26	76.04
15	1024.26	3	Water	0.22	34.49
16	600	3	Water	0.21	16.71
17	900	5	Water	0.16	28.04
18	300	5	Water	0.46	26.93
19	600	3	Ethanol	0.13	51.82
20	600	3	Water	0.43	37.82
21	175.74	3	Water	0.31	34.49
22	900	1	Ethanol	0.18	73.60
23	600	5.83	Ethanol	0.04	88.04
24	600	3	Ethanol	0.17	82.93
25	600	0.17	Water	0.11	15.60
26	600	3	Water	0.27	20.71

Bold values indicate statistically significant. **indicates highly significant.

**Table 4:** Analysis of variance of quadratic model for quercetin.

Source	Sum of squares	df	Mean square	F-value	*p*-value	Remarks
Model	18021.01	8	2252.63	10.41	**<0.0001** ^**^	Significant
A	344.92	1	344.92	1.59	0.2238	
B	65.26	1	65.26	0.3016	0.5900	
C	16810.30	1	16810.30	77.68	**<0.0001** ^**^	
AB	323.71	1	323.71	1.50	0.2380	
AC	370.13	1	370.13	1.71	0.2083	
BC	37.84	1	37.84	0.1749	0.6811	
A^2^	67.94	1	67.94	0.3139	0.5826	
B^2^	4.10	1	4.10	0.0190	0.8921	
Residual	3678.76	17	216.40			
Lack of fit	1107.30	9	123.03	0.3828	**0.9129** ^**^	Not significant
Pure error	2571.46	8	321.4			
Cor total	21699.77	25				

Bold values indicate statistically significant. **indicates highly significant.

#### 3.1.3 Model fitting

For the optimization of extraction procedure for maximum amount of quercetin and the evaluation of different variables on quercetin extraction, central composite design (CCD) was implemented. Three independent variables (time of irradiation, microwave power, type of solvent) were studied for two responses (amount of extract, amount of quercetin). The data recorded is given in Table 3.

ANOVA was used to analyze the data obtained after recording the responses for each run. Table 4 gives complete description of the results of ANOVA. From Table 4, it can be concluded that the type of solvent (C) significantly impacts the amount of quercetin extracted (*F* = 77.68, *p*-value <0.001). Other factors, i.e., microwave power (A) and irradiation time (B), also influence quercetin yield to some extent but the influence is not statistically significant (*F* = 1.59, *p*-value = 0.2238 and *F* = 0.3016, *p*-value = 0.5900 respectively). Interaction effects between the variables; power level and time of irradiation (AB) (*F* = 1.50, *p*-value = 0.2380), power level and solvent (AC) (*F* = 1.71, *p*-value = 0.2083), time of irradiation and solvent (BC) (*F* = 0.1749, *p*-value = 0.6811), as given in Table 4, were also statistically insignificant suggesting no synergistic effect of variables on the yield of quercetin. Additionally, the quadratic effect of power level (A^2^) and time of irradiation (B^2^) was also seen to be statistically insignificant (*F* = 0.3139, *p*-value = 0.5826 and *F* = 0.0190, *p*-value = 0.8921 respectively), indicating no curvilinear relationship among the tested range of variables.

Large *F*-value (10.41) and small *p*-value (0.01) indicate that the model is significant. The model’s lack of fit value (0.38) was not statistically significant as it was greater than 0.05. This insignificant lack of fit value indicates good predictability of the model. The results showed accuracy and predictability of the model.

According to the fit statistics, R2 value demonstrates the level of correspondence between experimental and predicted values of the model. Good fitness of the model can be judged by an R2 value closer to 1. A value of 0.8305 indicates that 83.05% of the variation occurring in amount of quercetin can be explained the variables used in design matrix of the experiment. The adjusted R2 and predicted R2 for the model were 0.7507 and 0.6547, respectively. Since the difference between Predicted R^2^ and Adjusted R^2^ is less than 0.2, it can be concluded that the values of the actual experiment are in reasonable agreement with the predicted values.

Equations can be used to make predictions about the tested response. Typically, the high levels of factors are coded by +1 whereas the low levels are coded as −1. Equations in terms of coded and actual factors are given below. The coded equation can also be used to identify the relative impact of factors by comparing their coefficients.


(1) $$\begin{array}{l} \text{Quercetin}=+50.67+4.64A+2.02B-25.43C+6.36\mathrm{AB}-\\ 4.81\mathrm{AC}+1.54\mathrm{BC}+2.21A^{2}+0.5431B^{2} \end{array}$$

(2) $$\begin{array}{l} \text{Quercetin}=C\;(\text{ethanol})+85.61012-0.029760\;\text{power level}-\\ 6.93482\;\text{time}+0.010602\;\text{power level}*\text{time}+\\ 0.000025\;\text{power leve}l^{2}+0.135764\;\mathrm{tim}e^{2} \end{array}$$

(3) $$\begin{array}{l} \text{Quercetin}=C\;(\text{water})+49.38066-0.061824\;\text{power level}-\\ 0.061824\;\text{time}+0.010602\;\text{power level}*\text{time}+\\ 0.000025\;\text{power leve}l^{2}+0.135764\;\mathrm{tim}e^{2} \end{array}$$

Equation 1 shows that the amount of quercetin obtained increases with increase in parameter A (power level), B (irradiation time), interaction effect AB (between microwave power and irradiation time), interaction effect BC (between irradiation time and type of solvent), quadratic effect A2 and quadratic effect B2. On the other hand, a negative relation between amount of quercetin and parameter C (type of solvent) and interaction effect AC (between microwave power and type of solvent) has been observed. The interaction factor AB possess the highest number (6.36) among all parameters. Thus, interaction between microwave power and irradiation time had the most significant effect on the amount of quercetin. However, the effect is not statistically significant (*p* = 0.2380, >0.05).

**Figure 1:**
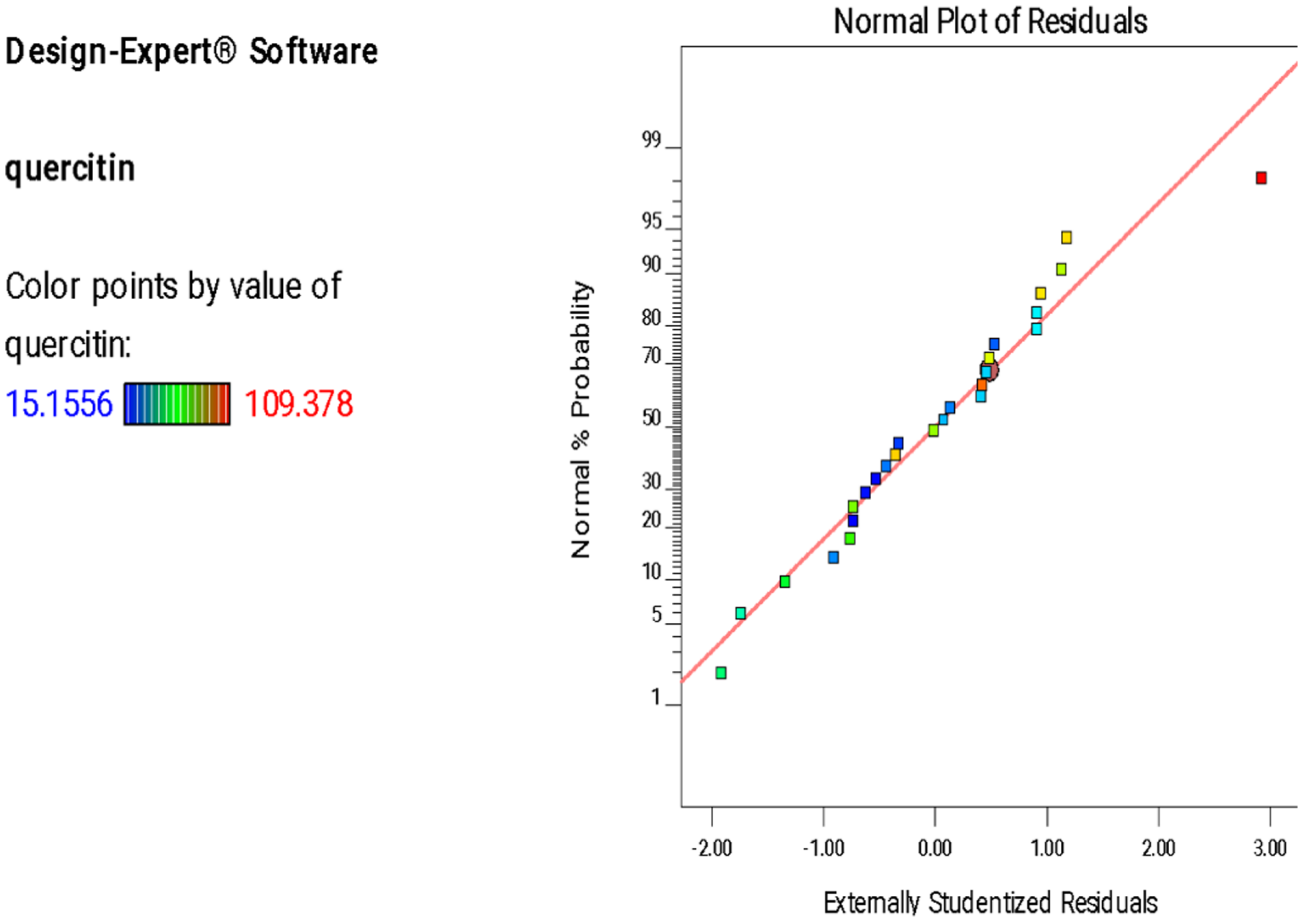
Normal plot of residuals for quercetin.

A normal plot of residuals was employed to assess the normality in residuals. Quercetin yield is represented by different colored points. The plot demonstrated the alignment of residuals against a diagonal red line. As shown in Figure 1, majority of the residuals lie close to the diagonal line indicating a normal distribution. Although a few outliers can be observed but the overall linear trend supports reliability and accuracy of the model.

#### 3.1.4 Significance of MAE parameters in terms of quercetin extraction

Table 4 represents the effect of microwave power, time of irradiation, type of solvent, their interaction effects and quadratic effects on the amount of quercetin extracted from red onions. From Table 4, it can be concluded that the type of solvent significantly impacts the amount of quercetin extracted (*p*-value < 0.001). Other factors also influence quercetin yield to some extent but the influence is not statistically significant.

**Figure 2:**
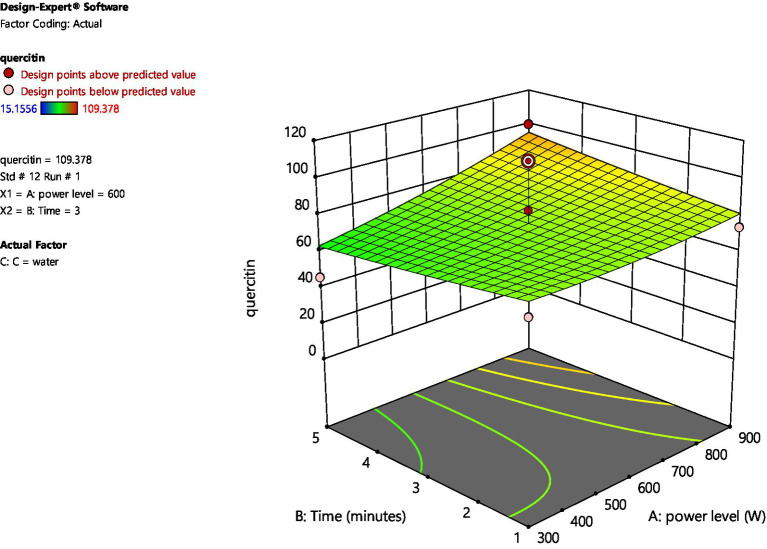
3D plot for amount of quercetin between Power level and Time (water).

**Figure 3:**
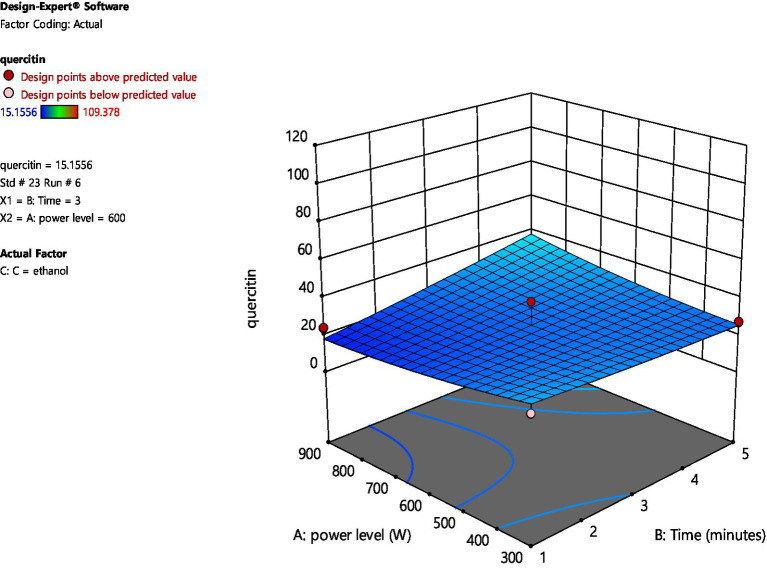
3D plot for amount of quercetin between Power level and Time (ethanol).

Figures 2, 3 represent 3D plot surfaces demonstrating relationship between power level, time of irradiation and amount of quercetin for water and ethanol, respectively. Figure 2 shows that as we increase power level from 300 to 900 W and time from 1 to 5 min, the amount of quercetin also increases. However, both these variables are not statistically significant.

On the other hand, when ethanol is used as a solvent, increasing microwave power and irradiation time had a very minimal increase in the amount of quercetin. The data points above and below the predicted values (run no. 1 and 6) are considered outliers and are further analyzed for the amount of quercetin through High Performance Liquid Chromatography (HPLC).

#### 3.1.5 Quantification of quercetin using HPLC-UV

**Figure 4:**
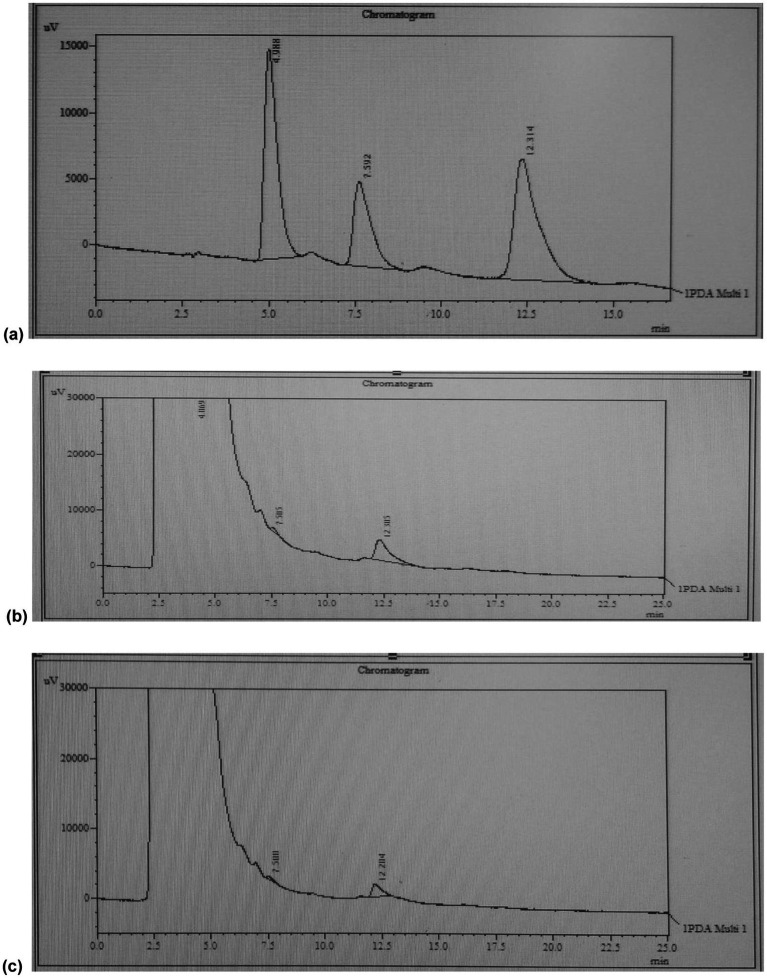
HPLC-UV chromatograms for **(a)** standard, **(b)** extract of run no.1 (water), and **(c)** extract of run no. 6 (ethanol).

HPLC was performed on the extracts from run no. 1 and 6. The chromatogram of the extract of run no. 1 revealed a peak at the retention time consistent with the standard of quercetin used (Figure 4). In contrast, the sample of extract of run no. 6 showed no peak consistent with quercetin standard thereby suggesting the absence of the compound in the sample (Figure 4).

A formula integrating the peak area of sample and standard was used to calculate the amount of quercetin in the sample. The formula used is given below:


$$\begin{array}{l} \text{Concentration of sample}=\frac{\text{peak area of sample}}{\text{peak area of standard}}\\ {}*\text{concentration of standard} \end{array}$$

Sample of extract of run no. 1 revealed a concentration of 86.10 mg of quercetin per gram of red onion extract which is equivalent to an amount of 32.07 mg of quercetin per gram of dry onion powder.

### 3.2 Animal trial

#### 3.2.1 Body weight

Body weight was monitored each week for weight gain. At the end of the study, weight of all rats was measured before the blood and liver sampling. Mean weight ranges of all groups namely control, HFD group, G1, G2 and G3 recorded at the end of the experimental study were 224.60 ± 20.06, 251.40 ± 13.4, 235 ± 18.04, 237.40 ± 29.27 and 233.6 ± 3.50, respectively. Body weight of rats in treatment groups is seen to be reduced as compared to HFD group but the results were not statistically significant (*p*-value 0.297).

#### 3.2.2 Biochemical analysis

**Figure 5:**
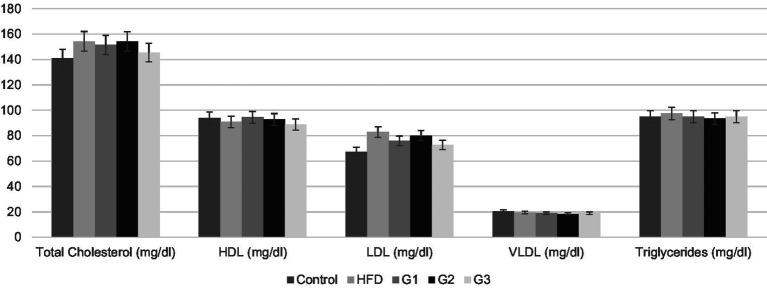
Graphical representation of mean serum Lipid profile parameters for Control, HFD, G1, G2, G3 group.

**Figure 6:**
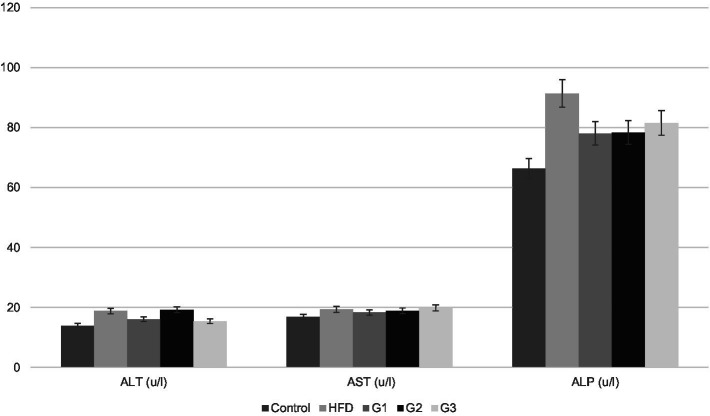
Graphical representation of serum levels of liver enzymes for Control, HFD, G1, G2, G3 group.

Levels of serum lipoproteins and liver enzymes were analyzed at the termination of study to check the efficacy of the synbiotic combination. Graphical representation of mean values of each parameter is given in Figures 5, 6.

#### 3.2.3 Serum lipid profile

##### 3.2.3.1 Total cholesterol

Figure 5 shows graphical representation of mean values serum cholesterol levels of all the groups. The mean serum cholesterol levels for control, HFD group, G1, G2 and G3 were 140.92 ± 4.37, 154.33 ± 6.45, 151.58 ± 6.34, 154.26 ± 3.40 and 145.48 ± 2.28, respectively. When compared with the HFD group (154.33 ± 8.17), mean cholesterol value of the therapeutic group 3 (G3) showed significant lower values (145.48 ± 2.89). *p*-value of 0.001 indicates significant difference between groups. Post-hoc analysis revealed significant difference between HFD and G3 with a *p*-value of 0.009 (*p*-value < 0.05).

##### 3.2.3.2 HDL cholesterol

As shown in Figure 5, HDL levels of group 1 (G1) showed an upward trend compared to HFD group but one-way analysis results’ were not statistically significant (*p*-value 0.269). No significant improvement was observed in HDL values of all groups. The mean serum HDL levels for control, HFD group, G1, G2 and G3 were 93.93 ± 2.57, 90.90 ± 1.71, 94.54 ± 2.57, 92.72 ± 4.8 and 91.71 ± 0.99, respectively.

##### 3.2.3.3 LDL cholesterol

The mean serum LDL cholesterol levels, as represented in Figure 5, were 67.45 ± 2.71 for control group, 82.92 ± 8.18 for HFD group, 76.02 ± 4.51 for treatment group 1 (G1), 80.02 ± 8.76 for treatment group 2 (G2) and 72.75 ± 3.24 for treatment group 3 (G3). Results of one way ANOVA revealed a significant difference between all groups (*p*-value 0.006, *p* < 0.05). Further *post hoc* analysis shows significant difference between HFD and G3 with a *p* value of 0.015. This means the treatment dose in group 3 was effective in lowering serum LDL levels in NAFLD rats.

##### 3.2.3.4 VLDL cholesterol

The mean values of VLDL cholesterol of all groups, as represented in Figure 5, are 20.46 ± 0.59, 19.49 ± 0.93, 18.98 ± 0.35, 18.47 ± 2.10 and 18.98 ± 0.08 for control, HFD, G1, G2 and G3, respectively. *p*-value of 0.08 depicts no significant difference between all groups. No improvement in VLDL levels was shown.

##### 3.2.3.5 Serum triglycerides

The mean serum triglycerides for control, HFD group, G1, G2 and G3 were 94.94 ± 1.56, 97.46 ± 0.00, 94.93 ± 1.78, 93.40 ± 10.61 and 94.93 ± 0.44, respectively. One way variance results’ showed no significant difference between groups (*p*-value 0.77).

#### 3.2.4 Liver enzymes

##### 3.2.4.1 Serum ALT

Figure 6 shows graphical representation of mean values of serum ALT of different groups. Mean ALT levels for control, HFD group, G1, G2 and G3 were 13.97 ± 0.00, 18.85 ± 2.10, 16.34 ± 2.65, 19.26 ± 1.67 and 15.46 ± 0.35, respectively. Significant difference between groups was observed (p-value 0.00). Mean serum ALT values for G2 (16.34 ± 2.65) and G3 (15.46 ± 0.35) decreased in comparison to HFD (18.85 ± 2.10). Post-hoc analysis revealed significant difference between G2, G3 and HFD with a p-value of 0.03 for G2 and a p-value of 0.005 for G3.

##### 3.2.4.2 Serum ALP

Serum levels of ALP of each rat was noted and mean values were calculated for each group. Graphical representation of mean serum ALP values of all groups is given in Figure 6. Mean values of all treatment groups decreased when compared to HFD group (91.37 ± 27.00). G1 had a mean of 78.09 ± 9.10, G2 78.38 ± 16.83 and G3 81.53 ± 13.42; however, there was no significant difference between the means of the group (*p*-value 0.240).

##### 3.2.4.3 Serum AST

Mean serum AST levels for control, HFD group, G1, G2 and G3 were 16.87 ± 0.00, 19.35 ± 3.86, 18.36 ± 1.05, 18.90 ± 2.48 and 19.85 ± 0.00, respectively. No improvement was observed in AST levels in any of the treatment groups in comparison to HFD group (*p*-value 0.246).

#### 3.2.5 Liver histopathology

**Figure 7:**
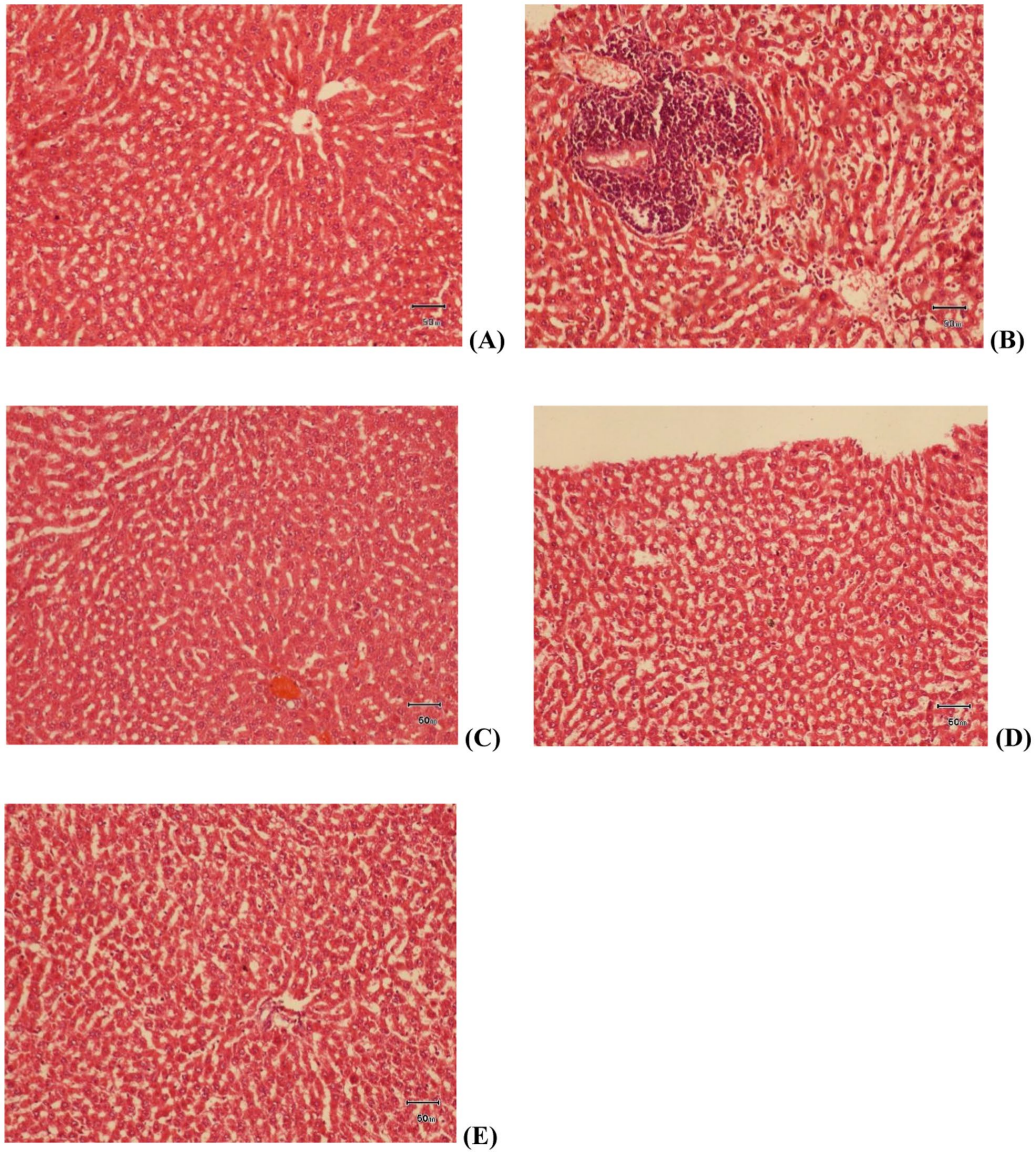
Histopathological slides of Liver tissue at 40X; slide **(A)** control group, slide **(B)** HFD group, slide **(C)** treatment group 1 (G1), slide **(D)** treatment group 2 (G2), slide **(E)** treatment group 3 (G3).

Once the rats were euthanized and dissected at the end of the study, liver samples were taken for histopathological study. Samples were taken from all groups namely control, high fat diet (HFD), treatment group 1 (G1), treatment group 2 (G2) and treatment group 3 (G3). Liver samples were stored in formalin, fixed with paraffin wax, dyed with H&E stains and then the slides were observed under electron microscope on 40X magnification. As represented in Figure 7, slide A (control group) shows normal liver tissue with no change in the hepatic cords. Slide B (HFD) shows mild steatosis, hepatocytes are misshapen and elongated. A tissue area of mononuclear infiltration representing inflammation can also be seen. Treatment group 1 (G1) is shown in slide C. Mild cellular swelling is observed in hepatocytes in this group. Slide D shows liver samples from G2. Almost normal appearance suggests somewhat improvement in steatosis and inflammation in this group. Lastly, treatment group 3 is represented by slide E. Mild cellular swelling in hepatocytes represent inflammation and no improvement.

## 4 Discussion

Traditional extraction techniques require large solvent amounts and extended extraction times, which often leads to the degradation of heat-sensitive compounds. Microwave-assisted extraction, however, presents a superior alternative by directly heating the sample through microwaves which increases the solubility of the target compound into the solvent, thus efficiently extracting the compound of choice. This makes it a faster, cheaper and environmental friendly technique by saving solvent, time and energy (15, 16).

Several factors impact the extraction efficiency in MAE. It includes properties of the solvent, such as its composition and polarity, alongside variables like volume, duration of exposure, microwave settings, system characteristics, temperature, and method application (23). Adjusting these parameters can improve extraction, such as González-de-Peredo et al. (24) found that a pH of 2, 93.8% methanol in water, 50°C extraction temperature, and 0.2:17.9 g: mL sample-solvent ratio showed maximum flavonol extraction, with solvent composition (concentration of methanol in water) as a significant parameter for extraction (*p* value less than 0.05). Similarly, González-de-Peredo et al. (25) employed Box–Behnken experimental design for optimization MAE parameters for maximum phenolic compounds. They identified 100% pure methanol as the solvent, maintaining a pH level of 2, operating at a temperature of 57°C, and employing a sample-solvent ratio of 0.2:8.8 g/mL as the ideal parameters for extracting total phenolic compounds from onion bulbs.

There is less research available on the extraction of a specific flavonoid compound and its optimization procedure. The present study however, focused solely on the extraction of quercetin from red onions. The results of this study indicate that the parameter type of a solvent significantly impacts the amount of the bioactive compound quercetin. 600 W microwave power, 3 min time of irradiation and using water as a solvent resulted in 32.7 mg of quercetin yield per gram of red onion powder. These findings are in accordance with the previous studies such as Kwak et al. (26) who reported a similar yield of quercetin from red onions using ultrasonic extraction technique. They aimed to quantify various quercetin glycosides in different onion varieties by using a combination of methanol, formic acid and water in a ratio of MFW; 50:5:45; v/v/v as a solvent. The results revealed that quercetin was the predominant compound in red onions with a concentration of 32.21 mg/g of dry weight of red onions. Similarity in results may be attributed to using water as a solvent in a combination with methanol.

Differences in quercetin yield reported in different studies are based on the variety of onion used, where and how it is grown, the part of onion used for extraction (flesh, skin, whole) as well as the method of extraction opted. For example, a study performed by Sagar et al. (27) on onion skin used ultrasound assisted extraction technique for extraction. 1 g onion skin powder was mixed with 25 mL methanol and was left overnight at 5oC. The samples were then sonicated for 10 min and afterwards vortexed for 5 min. After centrifugation the supernatant was collected for further analysis. Hplc analysis reported quercetin at a concentration of 11.8 mg/g in onion skin which is less than the results reported in this study. Similarly, Umer et al. (28) employed conventional maceration in an 80:20 v/v methanol-to-water to extract quercetin from red onions. HPLC results revealed a quantity of 26.69 mg/100 g of onions.

In addition to the extraction of quercetin, this study highlights the therapeutic potential of a synbiotic combination in the management of NAFLD. Due to the novelty of the concept, there is not much research available against synbiotic combination for the treatment of NAFLD. However, the results of the present study are validated by some studies examining the effect of quercetin and lactobacillus alone against NAFLD. The 2018 study performed by Liu et al. (29) on NAFLD induced adult mice disclosed that quercetin ameliorated lipid metabolism disorder and significantly reduced steatosis. Mice were administered a dose of 100 mg of quercetin per kg, as in the present study but for 10 weeks. Similarly, Saleh Al-Maamari et al. (30) investigated the effect of quercetin on SREBP-1c mRNA expression in mice fed with a high fat diet. Quercetin was administered at doses of 50 mg/kg and 100 mg/kg body weight for 28 days. Results showed that quercetin improved liver cell injury at both doses.

The results of a study investigating the effect of Lactobacillus species on non-alcoholic fatty liver disease (NAFLD) found that administered in rats at a dose of 10^9 CFU/g for 10 weeks attenuates the progression of NAFLD. Serum and liver analysis concluded that Lactobacillus treatment results in lower cholesterol levels and amelioration of liver steatosis (21). In a similar study, high fat diet fed rats received 10^9 CFU/day of *Lactobacillus acidophilus* KLDS1.0901 for 8 weeks via oral gavage, using 0.2 mL of sterile PBS. A significant reduction in body weight was observed. Additionally, a marked decrease in the serum levels of total cholesterol, LDL, total triglycerides, and liver enzymes was also seen (31).

In the present study, a 3-week short period administration of the combination of quercetin and lactobacillus significantly lowered serum cholesterol and liver enzymes suggesting their synergistic effect to be more potent than their effect alone. It can be concluded that when combined with quercetin, the effect of lactobacillus became more significant. These findings open doors for further investigation into the use of synbiotics as a treatment strategy for NAFLD.

## 5 Conclusion

The optimal conditions of MAE as generated by RSM are 600 W microwave power, 3 min time of irradiation and distilled water as a solvent. These findings offer crucial understanding of MAE optimization for extracting quercetin and may help future researchers and pharmaceutical industry to efficiently extract quercetin from red onions for its various therapeutic benefits. In addition, 3 weeks’ efficacy trial of the synbiotic combination reports significant changes in total cholesterol, LDL cholesterol and serum ALT only in treatment group 3 at a dose of 100 mg quercetin per kg BW + 10^9^ CFU/0.2 mL PBS of *Lactobacillus acidophilus*. Longer trials may reveal significant changes in other parameters that were not apparent in this study and can support the use of this combination as an effective treatment strategy against NAFLD.

## Data Availability

The raw data supporting the conclusions of this article will be made available by the authors, without undue reservation.
